# Integrated Systems Biology Analysis of Transcriptomes Reveals Candidate Genes for Acidity Control in Developing Fruits of Sweet Orange (*Citrus sinensis* L. Osbeck)

**DOI:** 10.3389/fpls.2016.00486

**Published:** 2016-04-08

**Authors:** Dingquan Huang, Yihong Zhao, Minghao Cao, Liang Qiao, Zhi-Liang Zheng

**Affiliations:** ^1^Plant Nutrient Signaling and Fruit Quality Improvement Laboratory, National Citrus Engineering Research Center, Citrus Research Institute, Southwest UniversityChongqing, China; ^2^Division of Biostatistics, Department of Child Psychiatry, New York University Langone Medical Center, New YorkNY, USA; ^3^Department of Biological Sciences, Lehman College, City University of New York, BronxNY, USA

**Keywords:** Citrus, orange, fruit, acidity, citrate, transcriptome, gene networks

## Abstract

Organic acids, such as citrate and malate, are important contributors for the sensory traits of fleshy fruits. Although their biosynthesis has been illustrated, regulatory mechanisms of acid accumulation remain to be dissected. To provide transcriptional architecture and identify candidate genes for citrate accumulation in fruits, we have selected for transcriptome analysis four varieties of sweet orange (*Citrus sinensis* L. Osbeck) with varying fruit acidity, Succari (acidless), Bingtang (low acid), and Newhall and Xinhui (normal acid). Fruits of these varieties at 45 days post anthesis (DPA), which corresponds to Stage I (cell division), had similar acidity, but they displayed differential acid accumulation at 142 DPA (Stage II, cell expansion). Transcriptomes of fruits at 45 and 142 DPA were profiled using RNA sequencing and analyzed with three different algorithms (Pearson correlation, gene coexpression network and surrogate variable analysis). Our network analysis shows that the acid-correlated genes belong to three distinct network modules. Several of these candidate fruit acidity genes encode regulatory proteins involved in transport (such as AHA10), degradation (such as APD2) and transcription (such as AIL6) and act as hubs in the citrate accumulation gene networks. Taken together, our integrated systems biology analysis has provided new insights into the fruit citrate accumulation gene network and led to the identification of candidate genes likely associated with the fruit acidity control.

## Introduction

Fruit is a specialized organ for flowering plants. Fully ripe fleshy fruits are rich in compounds important for human nutrition (such as sugars, vitamins, and antioxidants) and sensory traits (such as organic acids and secondary products responsible for attractive taste and flavor). Among the major compounds in fruits, sugars and organic acids are considered very important contributors for the taste trait, with the sugar/acid ratio recognized as the major determinant of fruit sweetness and ripeness (Albertini et al., 2006; Etienne et al., 2013; Osorio et al., 2013). Because increasing sugar contents in some fruits raises a major concern for human health, manipulating fruit sweetness by altering organic acid concentrations, either genetically or through management practice, has increasingly become a priority for improving fruit quality (Etienne et al., 2013). Therefore, significant efforts have been targeted towards mechanistic understanding of acid accumulation in fruits and ultimately breeding or selection for the fruit varieties with varying acid levels.

To reach the fully ripe stage for human consumption, fruits across species in general undergo three stages of development: cell division (characterized by slow fruit growth; Stage I), cell expansion (rapid fruit growth; Stage II) and fruit ripening (cessation of fruit growth and peak of active biochemical reactions for flavor; Stage III). Based on the respiration pattern and ethylene biosynthesis during the ripening stage, fruits are in general categorized into two physiological groups, climacteric fruits including tomato, apple, peach and banana, and non-climacteric fruits such as such as citrus, strawberry, grape and pepper (Gapper et al., 2013; Osorio et al., 2013).

Many studies of metabolic profiling during three stages of fruit development have led to better understanding of organic acids accumulation (Etienne et al., 2013). First, fruits from different species have different acid compounds. For example, citrate is predominant in citrus and strawberry, malate is dominant in apple, and tomato contains both citrate and malate although the content of citric acid is slightly higher than malic acid (Cercos et al., 2006; Zhang et al., 2011; Etienne et al., 2013; Morgan et al., 2013). Second, it appears that citric acid in citrus, tomato, strawberry and pepper fruits starts to accumulate during the transition from Stage I to Stage II and reach the peak at the late Stage II before it starts to decline during Stage III (Albertini et al., 2006; Cercos et al., 2006; Osorio et al., 2011, 2012; Zhang et al., 2011; Etienne et al., 2013). For acid accumulation, citric acid is intensely transported into the vacuole which then becomes highly acidified, but at Stage III it is released from the vacuole and through the tricarboxylic acid (TCA) cycle converted sequentially into isocitrate, 2-oxoglutarate and glutamate (Cercos et al., 2006; Nunes-Nesi et al., 2013). Glutamate is then utilized through at least two pathways. One is conversion to glutamine for further utilization, and the other is the GABA (gamma-aminobutirate) shunt which converts glutamate into GABA and finally succinate (Cercos et al., 2006).

Accumulating genetic studies have started to shed some light in the control of acid transport and metabolism in fruits. The existence of many varieties with different fruit acid levels suggests that citrate and other organic acids are genetically controlled (Fang et al., 1997; Boudehri et al., 2009; Terol et al., 2010; Bai et al., 2012; Zhang et al., 2012; Morgan et al., 2013; Cohen et al., 2014; Sauvage et al., 2014). However, only the *PH* gene has been convincingly demonstrated to act in the control of acid accumulation in melon fruits, although its biochemical mechanism remains a mystery (Cohen et al., 2014).

Extensive studies of molecular biology and transcriptomics have led to a model of citrate transport and utilization in fruit acidity control. These studies have been performed mostly in tomato, a major climacteric fruit model system, and citrus, which forms the largest tree fruit industry and has the potential of becoming a model system for non-climacteric fruits. For citrate transport into the vacuole, a lemon proton pump most closely related to *Arabidopsis* Autoinhibited H^+^-ATPase Isoform 10 (AHA10) has been proposed to drive citrate out of the vacuole (Aprile et al., 2011). There is corroborating biochemical evidence for the involvement of two types of H^+^-ATPase in acidification in lemon fruits (Muller et al., 1996). Although there is no convincing evidence for the correlation of citrate synthase or transporter gene with acid accumulation in citrus and tomato (Etienne et al., 2013), one recent study indicated that a mutation in a malate transporter-like gene is associated with low fruit acidity in apple (Bai et al., 2012). Regarding citrate conversion to isocitrate, several studies have shown that expression of *Aco* genes, which encode several isoforms of the enzyme aconitase involved in converting citrate to isocitrate, is up-regulated in both tomato and citrus fruits. Furthermore, varieties of acidless orange or mandarin exhibit higher expression of *Aco1* and *Aco2* genes than varieties with normal acidity (Terol et al., 2010) and Aco enzyme inhibitor experiments showed the role of *Aco* genes in citrate accumulation (Degu et al., 2011). The most convincing evidence supporting a role for *Aco* control in acid accumulation is the observed increase of citrate and malate in transgenic tomato lines where *Aco3b* is suppressed via antisense RNA (Morgan et al., 2013). Support for possible involvement of the GABA shunt at Stage III comes from a prior transcriptomic profiling study in citrus, where fruits at all of three stages were subjected to microarray analysis, showing up-regulation of several genes encoding enzymes involved in various steps of the GABA pathway, in particular Glu decarboxylase (GAD) (Cercos et al., 2006). The negative correlation between *GAD1/2* expression and acid loss is confirmed by recent studies in lemon and orange fruits (Aprile et al., 2011; Liu et al., 2014).

In summary, results from these gene expression studies suggest a very interesting model for understanding acid decline during fruit ripening. However, it has been reported that at least in some of citrus acidless varieties a consistently very low citric acid level is maintained throughout fruit development compared to normally acidic varieties (Albertini et al., 2006), indicating that genetic factors play a role early at stage I or II. Therefore, it remains to be tested whether the proposed citrate utilization model mainly operating at Stage III could explain the genetic variations in fruit acid contents in tomato, citrus or other species.

As a first step towards dissection of genetic mechanisms underlying organic acid accumulation in non-climacteric fruits, we have carried out an RNA sequencing (RNA Seq)-based transcriptomic study in sweet orange fruits. Sweet orange genome has been sequenced (Xu Q. et al., 2013; Wu G.A. et al., 2014), making it an excellent model system for non-climacteric fruits. Fruits collected at two stages (close to the end of Stage I and the middle of Stage II) from four varieties, which show differential fruit acidity, were used in RNA Seq analysis, followed by integradated sysntems analysis including Pearson correlation analysis, the Weighted Gene Coexpression Network Analysis (WGCNA), and Surrogate Variable Analysis (SVA). Taken together, our results have revealed candidate acid-related genes likely associated with fruit acidity control in developing orange fruits.

## Materials and Methods

### Plant Materials

Four varieties of sweet orange (*C. sinensis* L. Osbeck), Newhall, Xinhui, Bingtang and Succari, were grown in China National Citrus Germplasm Repository managed in Citrus Research Institute of Chinese Academy of Agricultural Sciences/Southwest University, Chongqing, China. They were all grafted onto the same root stock Trifoliate orange (*Poncirus trifoliata* L. Raf., synonym *Citrus trifoliate* L.). Representative fruits at various stages of development were harvested. Exocarp (flavedo) and mesocarp (albedo) were carefully removed. The remaining pulp tissues (endocarp) were dissected into small segments, weighted, and then quickly frozen with liquid nitrogen before acid, sugar and RNA extractions. For comparison of 45 and 142 DPA, three biological replicates were used. Because of the differences in fruit sizes and acid levels across varieties between 45 and 142 DPA, different sampling strategies were used. For 45 DPA, each replicate contained five fruits which were mixed and aliquoted for sugar and acid measurement and RNA Seq analysis. For 142 DPA, each fruit was used as a replicate and sliced into aliquots for both sugar and acid measurement and RNA Seq for each variety.

### Measurement of Sugar and Titratable Acid Contents

Total titratable acid content was measured by following the 0.1 M NaOH-based titration method according to (Cercos et al., 2006), using a citric acid coefficient of 0.064. Sugar levels were determined using the Sucrose/D-Glucose/D-Fructose kit (R-Biopharm/Roche) developed based on enzymatic reactions by hexokinase, glucose-6-phosphate dehydrogenase, and phosphoglucose isomerase, and the sum of glucose, fructose and sucrose was presented as the total sugar content.

### RNA Extraction, Sequencing Library Construction, and Data Processing

Approximately 5–8 g of pulp tissues were used for total RNA extraction by following the ethanol- and LiCl-based protocol described elsewhere (Tao et al., 2004). Total RNA samples were sent to Beijing Genomics Institute (BGI) Tech Solutions Co. in Shenzhen, China, for library construction, sequencing, data preprocessing and gene mapping. In briefly, total RNA was first treated with DNase I to degrade any possible DNA contamination, followed by mRNA enrichment through the oligo(dT) magnetic beads. mRNA was then fragmented into short fragments of approximately 200 nucleotides, which were used for cDNA synthesis. After purification, the double stranded cDNA is subjected to end reparation and 3′-end single nucleotide A (adenine) addition and sequencing adaptors ligation. Finally, the fragments are enriched by PCR amplification and the resulting library products were sequenced via Illumina HiSeqTM 2000. Raw data, which were deposited into NCBI GEO database (accession number 78046), were preprocessed by removing adaptor sequences and/or low quality reads, resulting in clean reads data. These clean reads data were then mapped to the sweet orange reference sequences (version 2.0) published recently (Xu Q. et al., 2013) and maintained in the *Citrus sinensis* Genome Annotation Project^1^ using SOAP aligner/SOAP2 (Li et al., 2009). The gene expression level is calculated by using the RPKM (Reads Per kb per Million reads) method (Mortazavi et al., 2008).

### Analysis of Citrus Homologs in *Arabidopsis* and Gene Ontology Enrichment

The sweet orange proteins for the whole genome (Xu Q. et al., 2013) was input into the functional annotation website Mercator (Lohse et al., 2014) for prediction of the most closely related proteins in *Arabidopsis*, using the TAIR release 10 of *Arabidopsis* proteome with a BLAST-Cutoff of 80. Subsequently, GO terms were assigned to citrus genes using the GO terms for their corresponding counter parts in *Arabidopsis* (GAF version 2.0 updated on September 5, 2014). GO enrichment analysis was performed in the Gene Ontology Enrichment Analysis Software Toolkit (Zheng and Wang, 2008), using the hypergeometric test with the Yekutieli-based adjustment for multiple testing (FDR under dependency) and a cutoff of FDR at 0.05.

### Statistical Analysis of Differentially Expressed Genes

Methods implemented in EdgeR (Robinson et al., 2010) are used to identify differentially expressed genes between 45 and 142 DPA for each of four varieties. After removing genes with low counts (i.e., removing if the mean counts is less than 10), the quantile-adjusted conditional maximum likelihood method is used to estimate the negative binomial dispersion parameters. Then the exact test method for the negative binomial distribution (Robinson and Smyth, 2007) is used to identify differentially expressed genes. The methods we chose are developed deliberately for studies with very small sample sizes. We are interested only in those with at least twofold difference. Statistical significance of the tests is controlled at FDR of 0.05.

### Pearson Correlation Analysis

Pearson correlation coefficients (Pcc) are calculated between citric acid content and gene expression levels on three biological replicates. We focus only on 7,430 differentially regulated genes and the gene expression levels are log2 transformed to normalize the data distribution. The correlation is significant at level of 0.05 (FDR adjusted).

### Global Characterization of the Transcriptome Data via Clustering Analysis

Clustering analysis is an important unsupervised learning technique for data exploratory analysis. First, we use K-means clustering via principal component analysis (Ding and He, 2004) of all available gene expression data to assess the quality of our biological replicates and to find samples with more homogenous gene expression patterns. The hierarchical clustering analysis (Everitt et al., 2001) using average linkage with Pearson correlation-based distance is also used for this purpose (**Figure 2B**).

Second, to identify genes with similar expression patterns across samples from all four genotypes at both 45 and 142 DPA (**Figure 3A**; **Supplementary Figure S2**), we perform the average linkage- based hierarchical clustering analysis using 7,430 differentially regulated genes. Again, the Pearson correlation-based distance criterion is used.

### Network Construction and Visualization

Weighted gene co-expression network analysis (WGCNA) is a powerful and general approach for identifying which set of genes/functional pathways are linked to phenotypes. We constructed the networks by using functions from the WGCNA package in R (Langfelder and Horvath, 2008). Specifically, pairwise Pearson correlations for all possible pairs of 7,430 genes are calculated. Construction of a weighted gene network requires the choice of the soft thresholding power β (Zhang and Horvath, 2005). Here the power β is chosen such that the scale-free topology criteria *R*^2^ of the network reaches 0.9, which corresponds to a power of 24 in our data. The 7430^∗^7430 adjacency matrix is defined as *A* =[*a*_ij_], where a_ij_ = |cor(v_i_,v_j_)|^β^ with *v_i_* and *v_j_* as expression levels at log2 scale for genes *i* and *j*. The networks are exported to Cytoscape for visualization.

The modules (i.e., subsets of genes) with coherent expression profiles are detected using functions from the WGCNA package in R. First, the adjacency matrix *A* is transformed into the topological overlap matrix (TOM) Ω =[*W*_i,j_], where

$$W_{ij}=\frac{a_{ij}+\Sigma_{u}a_{iu}a_{uj}}{\min\{\Sigma_{u}a_{iu},\Sigma_{u}a_{uj}\}+1-a_{ij}}.$$

TOM provides a measure of similarity among genes (Zhang and Horvath, 2005). The average linkage hierarchical clustering with the topological overlap measure-based distance criterion is used for module detection. To identify modules that are significantly correlated with the acid contents, we calculate Pearson correlations between the acid content and the first eigengene of each module. The first eigengene is chosen because it is believed that they can provide optimal summaries of gene expression profiles for the given modules.

### Surrogate Variable Analysis

The SVA approach is also used to find the gene expression and acid level association. It is understood that many factors, such as batch effects and environmental and technical variations, might have a substantial impact on transcriptome profiling. If ignored, it may result in reduced study power and biased biological conclusion. To adjust for those unknown, unmodeled, or latent sources of noise, surrogate variables are first constructed using functions in the R package “sva” (Leek and Storey, 2007; Leek et al., 2012) and then controlled, along with genotype and developmental stage, in the subsequent regression analysis to identify a list of candidate genes associated with fruit acidity.

### Quantitative Reverse Transcription-PCR (qRT-PCR) Analysis

RNA was reverse transcribed after removal of DNA by the DNase-treatment. Gene-specific primers (**Supplementary Table S2**) were designed for qRT-PCR analysis using SYBRGreen. As described elsewhere (Xin et al., 2005), relative mRNA levels were determined by first normalizing their PCR threshold cycle numbers with those of the reference gene (Cs1g05000/Actin) and setting the relative mRNA levels for each gene in Newhall at 45 DPA (**Table 1**) or 142 DPA (**Supplementary Table S5**) as 1 or 100, respectively, depending on the genes.

**Table 1:** Pearson correlation analysis of gene expression values detected by RNA sequencing and qPCR.

Genes	Methods	45 DPA				142 DPA				Pcc	*p*-value
					
		Newhall	Xinhui	Bingtang	Succari	Newhall	Xinhui	Bingtang	Succari		
Cs1g16150	RNA seq	5.0	0.8	0.4	0.0	40.2	30.4	7.7	0.0	0.98	2.8E-05
(AHA10)	qPCR	1.0	0.2	0.1	0.0	17.0	10.3	5.7	0.0		
Cs1g20480	RNA seq	9.9	10.7	14.4	11.8	1.1	0.6	0.8	13.1	0.99	4.3E-06
(AIL6)	qPCR	1.0	1.2	1.4	1.2	0.1	0.1	0.1	2.0		
Cs5g31400	RNA seq	2.3	1.3	0.9	0.5	30.7	14.1	6.3	2.4	0.98	1.7E-05
(TT8)	qPCR	1.0	0.8	0.5	0.1	22.6	6.5	4.2	1.1		
Cs6g08410	RNA seq	1.0	0.7	0.6	0.5	11.2	6.9	2.1	0.5	0.94	5.8E-04
(APD2)	qPCR	1.0	0.6	0.5	0.7	29.8	19.0	11.7	1.3		
Cs9g17580	RNA seq	0.5	0.3	0.1	0.1	8.2	3.4	1.4	0.1	0.94	5.8E-04
(unknown)	qPCR	1.0	0.7	0.4	0.7	12.7	6.2	2.6	0.1		
Cs6g15800	RNA seq	0.7	1.4	1.2	1.8	5.2	13.3	21.8	26.0	0.99	4.2E-06
(glycosyltransferase)	qPCR	1.0	2.3	1.3	2.0	6.2	20.3	20.6	45.9		
Cs1g25820	RNA seq	0.0	0.0	0.2	0.0	2.2	0.2	0.8	18.7	0.98	1.5E-05
(Heavy metal-associated domain containing protein)	qPCR	1.0	1.1	0.9	2.1	10.2	3.1	6.2	135.6		


*Expression patterns for seven genes which were determined to be differentially regulated between different varieties by RNA sequencing (RNA seq) were validated by using quantitative PCR (qPCR) analysis of the RT products. Values are the means of RNA seq data (RPKM) or qPCR data (with the value for Newhall at 45 DPA set as 1 after normalization to the *Actin* control) from three biological replicates. DPA, days post anthesis; Pcc, Pearson correlation coefficient.*

## Results

### Analysis of Fruit Acidity at Early Stage of Fruit Development in Four Orange Varieties

**FIGURE 1:**
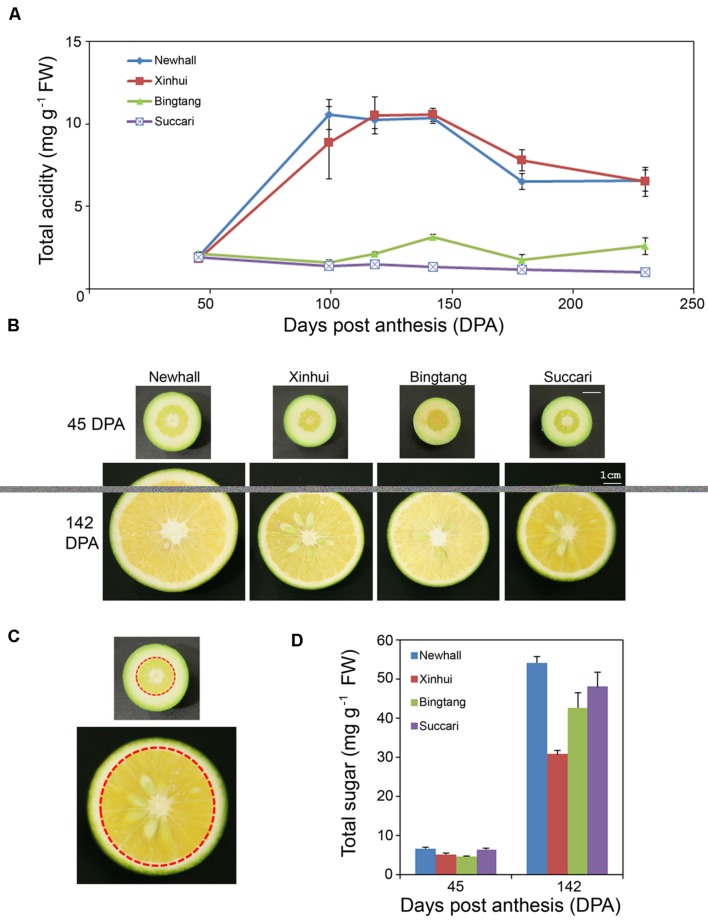
**Acid and sugar metabolic changes during orange fruit development.** **(A)** Total acidity in endocarps of four orange varieties. Six time points are 45, 99, 118, 142, 179, and 230 days post anthesis (DPA). FW, fresh weight. Values are means and SE of 3–4 fruits. **(B)** Images of half fruit of four orange varieties at 45 and 142 DPA. The same scale bar represents 1 cm for all fruits. **(C)** Images showing the endocarp fruit tissues (indicated by the areas within a red dotted circle) excised for metabolic and RNA analyses. Exocarp (albedo) and mesocarp (flavedo) outside the red circle were carefully removed. Endocarp or pulp botanically is the collection of carpels filled with juicy sacs or vesicles. **(D)** Total sugar contents in endocarps of four orange varieties at 45 and 142 DPA. Total sugar includes glucose, fructose and sucrose. Values are means and SE of three fruits.

To determine which stages of fruit development could be used for profiling transcriptomes related to citric acid accumulation in sweet oranges, we have selected two popular varieties with normal acid contents, Newhall and Xinhui, and two varieties with low acid levels, Succari and Bingtang, for analysis of total titratable acids content changes in the pulp tissues from fruits at 45, 99, 118, 142, 179, and 230 DPA. In citrus fruits, the majority of titratable acids measured are citrate, with malate only accounting for approximately 10%, and the titration results correlated well with measurement of individual acids by HPLC methods (Cercos et al., 2006). Our results showed that at 45 DPA (which is close to the end of Stage I of fruit development; **Supplementary Figure S1**), there was no obvious difference in acid content between the four varieties (**Figure 1A**). Fruit acid accumulated rapidly and reached the peak at 99–142 DPA (which corresponds to early and middle Stage II; **Supplementary Figure S1**) for Newhall and Xinhui. However, acid content declined at late Stage II (179 DPA), and stayed almost the same close to the end of Stage III (230 DPA). This acid accumulation pattern in Newhall and Xinhui is similar to earlier reports (Albertini et al., 2006; Cercos et al., 2006), consistent with the notion that organic acids in citrus fruits undergo the most dramatic and dynamic change at Stage II where cell expansion is predominant. In contrast, Bingtang and Succari exhibited different acid accumulation kinetics. Bingtang started to accumulate acids at 118 DPA and reached the peak at 142 DPA and then declined at 179 DPA, although at 230 DPA the acid content increased slightly. Interestingly, Succari did not show any acid accumulation throughout fruit development (**Figure 1A**). While Newhall has slightly larger fruits at 142 DPA, the other three varieties have very similar fruit sizes (**Figure 1B**). Sugar content measurement at 45 and 142 DPA showed that these four varieties all increased their sugar accumulation in mid Stage II (**Figure 1D**). Therefore, these four orange varieties mainly differ in the fruit acidity rather than sugar accumulation.

### Overview of Fruit Transcriptomes

As acid accumulation at 142 DPA is the major factor in determining fruit sweetness in the four varieties, we decided to extract RNA from the pulp tissues of fruits at 45 and 142 DPA (**Figure 1C**) for transcriptomic analysis. RNA sequencing was used to profile transcriptomes for eight genotype and stage combinations, each with three biological replicates. Mapping the sequence reads to the orange genome revealed that a total of 24,166 unique genes have perfect matches with the sweet orange genes. Those genes with an average of smaller than 10 raw counts in any of the eight fruit genotype-stage combinations were considered low abundant genes and thus were discarded, resulting in a total of 17,540 genes for statistical analysis. EdgeR (Robinson et al., 2010) was used to identify significantly expressed genes by comparing 142 vs. 45 DPA for each of four varieties, resulting in a total of 7,430 genes differentially expressed in any of these four varieties with a two-fold cutoff (**Supplementary Figure S1**). Gene expression levels for seven selected genes obtained through qRT-PCR were found to highly correlate with that of RNA seq, with Pearson correlation coefficients (Pcc) ranging from 0.94 to 0.99 and p-values of <0.001 (**Table 1**), indicating that our RNA sequencing data are reliable.

**FIGURE 2:**
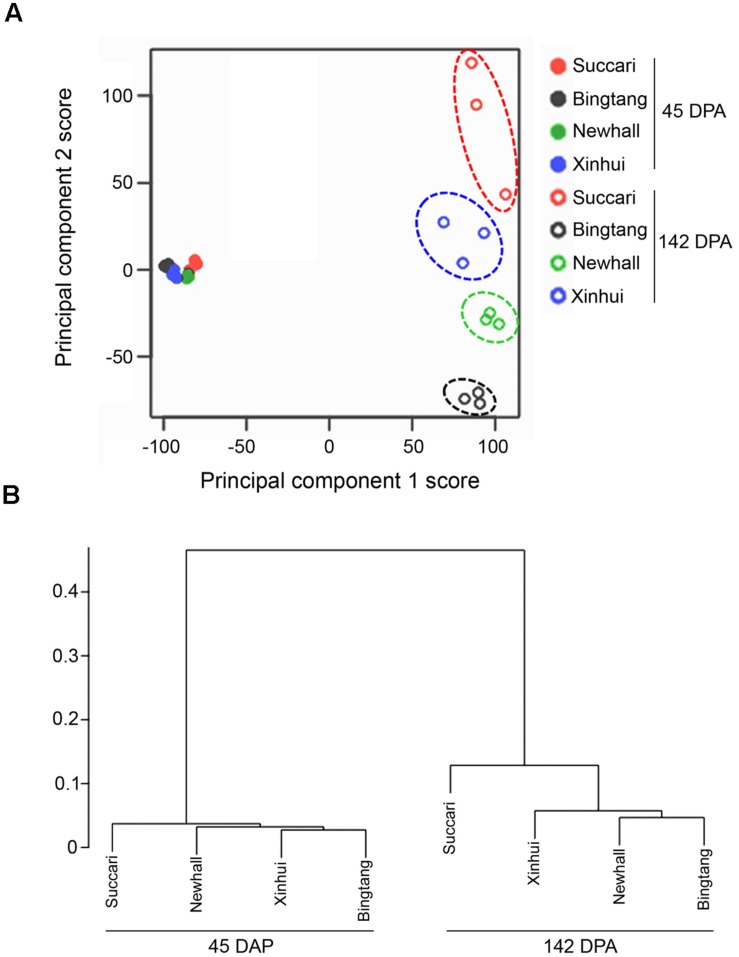
**Global relationships between four orange varieties and their biological replicates.** **(A)** Principal component analysis plot of RNA seq data. Expression of 17,540 genes in each biological replicate for every orange variety at 45 and 142 DPA (days post anthesis) were used for principal component analysis. The *x*- and *y*-axes represent the scores of principal components 1 (explained 48.7% of total variance) and 2 (11.2% of total variance), respectively. **(B)** Cluster dendrogram of transcriptome data using the mean expression level of three biological replicates in each of orange varieties for 7,430 differentially expressed genes.

K-means clustering via principal component analysis of 17,540 genes from those eight fruit variety-stage combinations clearly indicates that the 24 samples can be clustered into five groups (**Figure 2A**). The four orange varieties at 45 DPA have homogenous gene expression patterns. In contrast, each of the four orange varieties at 142 DPA shows rather distinct gene expression patterns (**Figure 2A**), although the variation for three biological replicates of Succari at this stage was slightly larger than that for the other three varieties. Overall, these assessments indicate that our biological replicates are reproducible. Using the mean expression level of three biological replicates in cluster analysis of 7,430 differentially regulated genes (**Figure 2B**), we found that expression profiles at 45 DPA do not differentiate among all four genotypes, while those at 142 DPA differentiate the four varieties. Moreover, at 142 DPA Newhall, Xinhui and Bingtang oranges are more similar to each other, while Succari is distinct from the other three genotypes.

**FIGURE 3:**
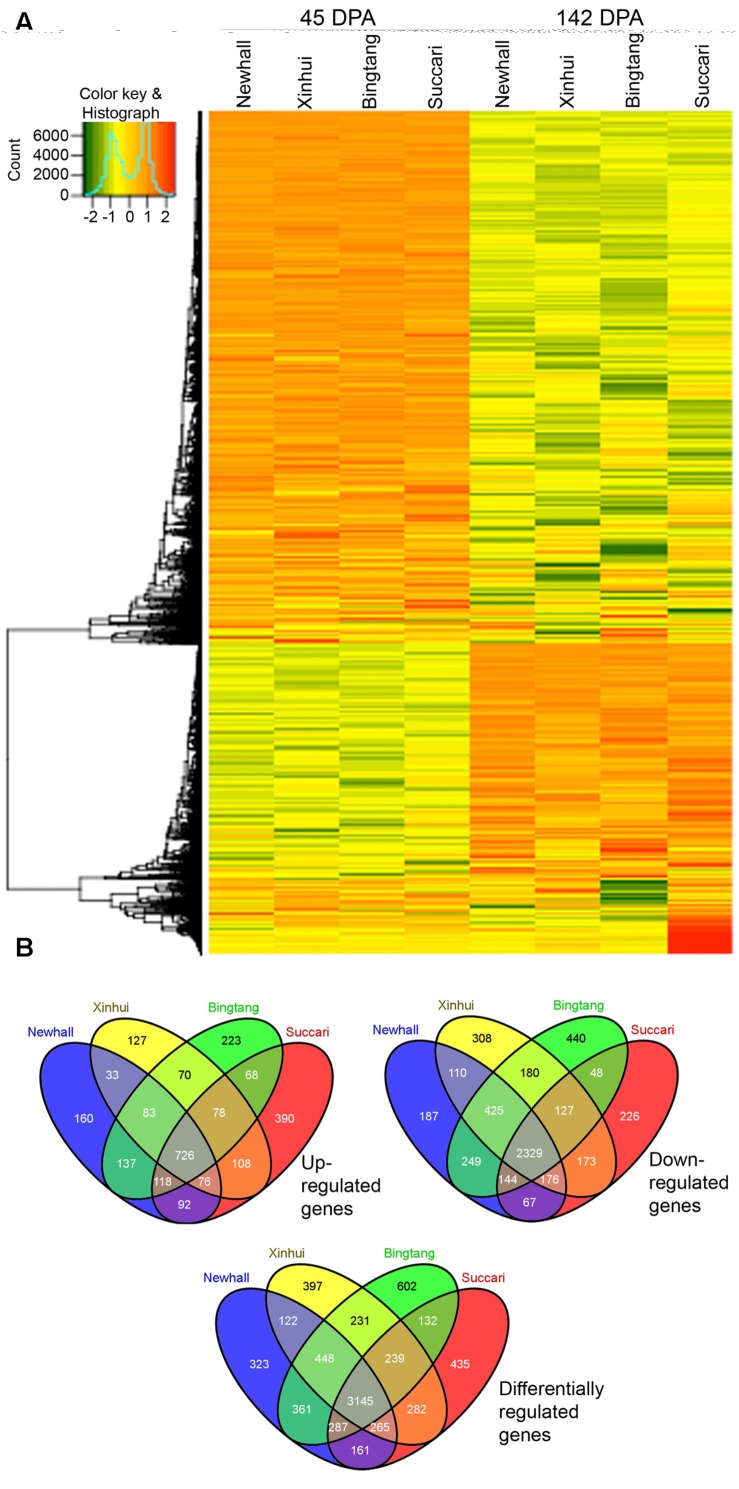
**Heatmap and Venn diagram of differentially expressed genes.** **(A)** Heatmap of 7,430 differentially expressed genes in orange fruits between 45 and 142 DPA (days post anthesis). **(B)** Venn diagram of up-regulated, down-regulated and differentially regulated genes from 45 to 142 DPA in four varieties.

Hierarchical clustering of 7,430 differentially regulated genes showed that the majority of genes exhibited similar expression patterns in the four varieties (**Figure 3A**, **Supplementary Figure S2** and **Table S1**). For example, the largest cluster (Cluster 1) has 4,482 genes which exhibit a similar down-regulation pattern when comparing expression at 142 with 45 DPA, while the second largest cluster (Cluster 2) has 2,055 genes that show a similar up-regulation pattern (Figure S2). This result indicates that from 45 to 142 DPA the majority of genes turn on and off similarly in these four orange varieties. Venn diagram analysis revealed that 726 and 2,329 genes were commonly up-regulated and down-regulated in all four varieties (**Figure 3B**). Together, there are 3,145 differentially regulated genes from 45 to 142 DPA that are common to all four varieties, indicating that this group of orange early fruit development-related genes are likely the most conserved ones in four sweet orange varieties.

### Pearson Correlation Analysis of Fruit Acidity and Gene Expression Changes

To identify the genes significantly correlated with the acid accumulations in orange fruits, pairwise correlation between gene expression and acid levels were performed using classical Pearson correlation analysis on three biological replicates. Among 7,430 differentially regulated genes, a total of 39 genes had Pcc larger than 0.80 (positive correlation) or smaller than -0.80 (negative correlation), with a minimal false discovery rate (FDR) of 1.0E-04 (**Table 2**). The majority (29 out of 39, approximately 75%) of these genes were positively correlated with acid level, with Cs6g08410 exhibiting the strongest correlation (Pcc = 0.96), which is mostly closed related to *Arabidopsis* APD2 gene, a RING/U-box superfamily protein shown to be involved in pollen development (Luo et al., 2012).

**Table 2:** A list of genes strongly correlated with the acid level in orange fruits.

CsGID	Pcc	FDR	AtGID	*Arabidopsis* gene description
**Metabolic process (10)**
Cs6g13410	-0.81	3.3E-04	At5g06390	FLA17, cell wall arabinogalactan protein 17 precursor
Cs9g18830	0.89	4.8E-06	At3g16520	UGT88A1, UDP-glucosyl transferase 88A1
orange1.1t00260	0.82	2.6E-04	At2g45510	CYP704A2, cytochrome P450
Cs5g03440	0.82	2.6E-04	At2g45970	CYP86A8, cytochrome P450
Cs2g19590	-0.88	9.2E-06	At3g26300	CYP71B34,cytochrome P450
Cs5g09970	0.81	3.6E-04	At4g22880	LDOX, proanthocyanin biosynthesis and vacuole formation
Cs2g19300	0.87	1.6E-05	At1g19640	JMT, Jasmonic acid carboxyl methyltransferase
Cs2g19320	0.93	2.9E-11	At5g66430	SAM-dependent methyltransferase
Cs3g23110	-0.80	6.4E-04	At3g16150	ASPGB1, asparaginase B1
Cs3g11790	0.85	3.9E-05	At4g24220	VEP1, steroid metabolic process, xylem and phloem pattern formation
**Transport** **(5)**				
Cs1g16150	0.89	2.4E-06	At1g17260	AHA10 (H^+^-ATPase), vacuolar biogenesis and acidification
Cs3g15070	0.88	9.1E-06	At1g15690	AVP1 (H^+^-PPase), apoplastic pH and auxin transport
Cs1g20080	0.93	5.8E-08	At1g07670	ECA4, calcium transport ATPase, calcium signaling
Cs5g13360	-0.80	6.4E-04	At1g78610	MSL6, mechanosensitive ion channel
Cs1g14330	0.84	7.6E-05	At4g19640	Ara7, Rab small GTPase, function in trafficking to vacuole
**Transcription factors** **(4)**
Cs1g20480	-0.80	6.4E-04	At5g10510	AIL6, AP2-domain transcription factor, development
Cs5g31400	0.89	2.4E-06	At4g09820	TT8, bHLH family, regulation of flavonoid pathways
Cs9g03070	0.90	1.7E-06	At3g13540	MYB5, seed coat development
orange1.1t04785	0.85	4.1E-05	At4g09460	MYB6, response to hormones gibberellin, jasmonic acid and salicylic acid
**Protein degradation** **(3)**
Cs2g04770	0.85	4.7E-05	At1g09580	XBCP3, Xylem bark cysteine peptidase
Cs3g15620	0.93	5.8E-08	At5g01450	APD2, RING/U-box superfamily protein, pollen development
Cs6g08410	0.96	6.5E-10	At5g01450	APD2, RING/U-box superfamily protein, pollen development
**Other** **(2)**
Cs8g02590	-0.82	2.2E-04	At4g35070	SBP, S-ribonuclease binding protein family
Cs2g15460	-0.81	3.3E-04	At3g58640	MAPKKK-related kinase, protein phosphorylation
**Unknown function** **(15)**
Cs1g10860	0.80	6.4E-04	No hit	
Cs1g26060	0.87	1.6E-05	At2g17710	Unknown
Cs2g05055	0.85	4.1E-05	No hit	
Cs2g21750	0.88	9.2E-06	No hit	
Cs3g25020	0.83	1.6E-04	No hit	
Cs4g19810	0.86	2.0E-05	At2g20740	Tetraspanin family protein
Cs5g25860	-0.81	3.1E-04	At3g26040	HXXXD-type acyl-transferase family protein
Cs5g32490	0.91	6.0E-07	No hit	
Cs6g15070	0.80	6.4E-04	At3g59320	Eukaryotic protein of unknown function (DUF914)
Cs7g17040	0.92	3.8E-07	No hit	
Cs9g03580	-0.84	6.7E-05	No hit	
Cs9g03065	0.94	3.9E-08	No hit	
Cs9g17580	0.93	7.6E-08	At3g49055	Unknown
orange1.1t01749	0.90	2.4E-06	At2g47115	Endomembrane system
orange1.1t03644	-0.83	1.7E-04	No hit	


*A total of 39 citrus genes are highly correlated with the acid levels in orange fruits of four varieties, with a Pearson correlation coefficient (Pcc) of at least ± 0.80 and an adjusted p-value (FDR, false discovery rate) of larger than 1.0E-04. CsGID, Cs gene ID. The number of genes for individual biological process is indicated in parenthesis. The most closely related homolog of *Arabidopsis* genes are presented as AtGID (At gene ID), with gene description shown. No hit, no *Arabidopsis* homolog for CsGID.*

Gene ontology (GO) analysis of these 39 acid-correlated genes did not reveal any significantly overrepresented GO. Although 15 of those genes were classified as unknown biological process, the remaining 24 genes can be classified into four major GO categories, metabolic processes, transport, transcription factors and protein degradation (**Table 2**). The metabolic process category contains 10 genes, among which three genes are closely related to *Arabidopsis* cytochrome P450-type genes (CYP704A2, CYP86A8, and CYP71B34). Other genes are involved in various aspects of metabolic process. For example, Cs2g19320 is closely related to *Arabidopsis* JMT involved in jasmonate synthesis. Cs2g19320 is highly similar to *Arabidopsis* SAM-dependent methyltransferase. Cs3g11790/VEP1 encodes an enzyme participating in steroid metabolic process and involved in xylem and phloem pattern formation (Jun et al., 2002; Bauer et al., 2012). Cs9g18830 is homologous to a member of UDP-glucosyltransferase, UGT88A1. It remains unclear whether the metabolic processes controlled by these gens relate to acid accumulation.

The GO category of transport, which has five genes, is of particular interest. Cs1g16150 is predicted to encode an H^+^-ATPase and most similar to *Arabidopsis* AHA10, which has been shown to function in vacuolar biogenesis and acidification (Baxter et al., 2005). Furthermore, this gene was also identified by comparing the transcriptomes using lemon, orange, and pummelo varieties with differential acid levels (Aprile et al., 2011; Shi et al., 2015), indicating that H^+^ pump activity regulation could be a potentially important mechanism in the control of fruit acidity. Another transport-related protein (Cs3g15070) is similar to *Arabidopsis* AVP1, which regulates apoplastic pH and auxin transport (Sarafian et al., 1992; Li et al., 2005). Two other genes, Cs1g20080 and Cs5g13360, are most closely related to *Arabidopsis* ECA4 (calcium transport ATPase) and MSL6 (a mechanosensitive ion channel), respectively (Wu et al., 2002; Haswell et al., 2008). In addition, Cs1g14330 is most closely related to *Arabidopsis* Ara7 which acts in trafficking to vacuole (Jia et al., 2013). Except for MSL6, these transport-related genes have very strong positive correlations with fruit acidity. Given that citrate movement into and storage in the vacuole is critical for acid accumulation, this result highly indicates a potential role for these transport-related genes in citrate homeostasis in orange fruits.

In addition, four transcription factor genes are highly correlated to acid level. Cs1g20480 is orthologous to *Arabidopsis* AIL6, an AP2-domain containing transcription factor. AIL6 is critical for auxin-mediated flower development (Krizek, 2009, 2011). Cs5g31400 is most closely related to *Arabidopsis* TT8, a member of a large family bHLH transcription factor. Given that TT8 is an important regulator of flavonoid biosynthesis pathway (Xu W. et al., 2013) and as described above, another proanthocyanin biosynthesis gene, Cs5g09970/LDOX, is also positively correlated with acid level, it is possible that flavonoid/anthocyanin metabolism may play a role in the control of acid accumulation in orange fruits. Two other transcription factors belong to the large MYB family, Cs9g03070 (MYB5), and orange1.1t04785 (MYB6) and they also show positive correlations with acid level.

Three genes are predicted to act in protein degradation based on GO analysis or functional demonstration in *Arabidopsis*. Cs2g04770 is similar to XBCP3, a vacuolar cysteine peptidase (Beers et al., 2004; Carter et al., 2004). The other two closely orange genes, Cs6g08410 and Cs3g15620, which have the strongest correlation with acid level (**Table 2**), are most closely related to *Arabidopsis Aberrant Pollen Development 2* (*APD2*). *Arabidopsis* APD2 acts in ubiquitin-mediated protein degradation and is involved in cell division during pollen development (Luo et al., 2012).

Two other genes also have been annotated with biological process, Cs2g15460 (encoding a MAPKKK-related protein kinase) and Cs8g02590 which is closely related to SBP (a member of S-ribonuclease binding protein family).

### Network Analysis of Early Fruit Development Transcriptomes Using WGCNA

**FIGURE 4:**
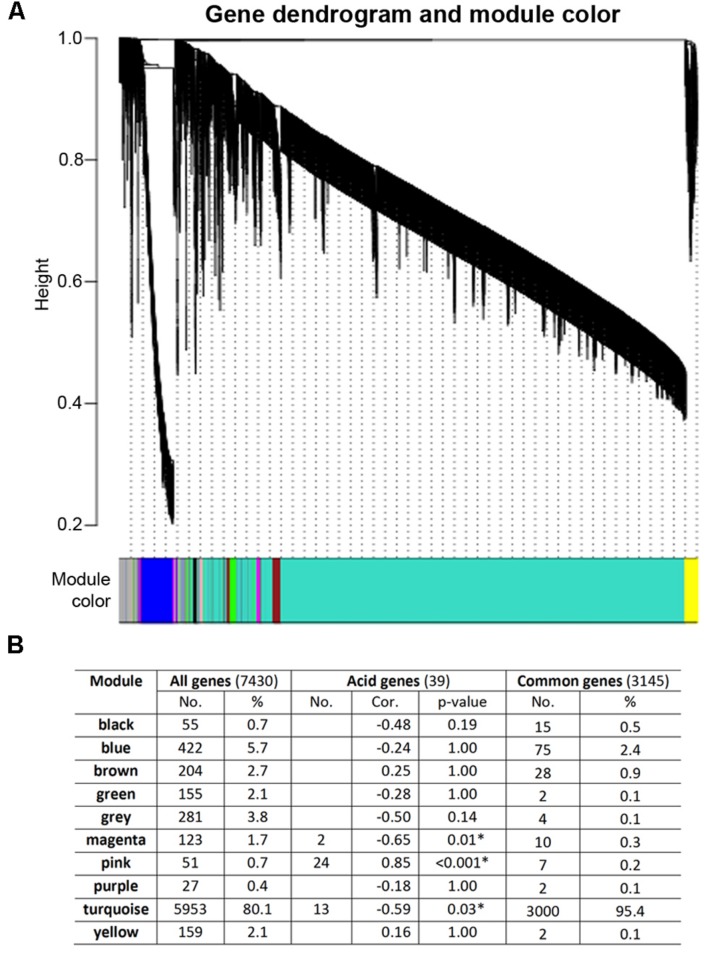
**Coexpression network analysis of early fruit development transcriptomes using WGCNA.** **(A)** Dendrogram and modules for 7,430 differentially expressed genes. The major tree branches represent 10 modules shown in different colors. Each leaf in the tree represents one gene. **(B)** Distribution of 7,430 differentially expressed genes and correlation of modules and acid levels. Number (No.) and percentage (%) of the genes for each module and correlation coefficient (Cor.) between acid contents and module eigengene expression were shown. *P*-value, FDR adjusted *p*-value. Asterisk indicates statistically significant correlation.

To gain further insights into the gene expression networks in early fruit development with a particular focus on the transcriptional architecture of citrate accumulation, WGCNA were used for gene coexpression network construction, module detection and visualization (Langfelder and Horvath, 2008). This tool has been widely used in animal systems analysis and recently used in fruit crops including coexpression network analysis in citrus and strawberry (Zheng and Zhao, 2013; Hollender et al., 2014). From the coexpression network constructed based on 7,430 differentially regulated genes between 45 and 142 DPA, we have identified a total of 10 distinct modules (**Figure 4A**). The majority (80%) of genes belongs to the largest module Turquoise, and each of the other nine modules contains only 0.4–5.7% of genes (**Figure 4B**). Furthermore, the most conserved early fruit development genes (3,145) are heavily distributed (95%) to the Turquoise module, but the 39 strongly acid correlated genes (**Table 2**) are distributed into three modules, Magenta (2 genes), Pink (24 genes), and Turquoise (13 genes). The Pink module is of particular interest as 24 of 39 acid related genes belong to this module, representing 47% of all the Pink module genes in the network (**Figure 4B**). Indeed, correlation analysis between the module eigengenes and fruit acid levels suggests that the Pink module is most strongly and positively correlated with acid level (Pcc = 0.85, adjusted *p*-value < 0.001), while the other two modules, Magenta and Turquoise, only showed weak and negative correlations with acid levels (**Figure 4B**).

### Analysis of Citrate-Correlated Modules and Subnetworks

**FIGURE 5:**
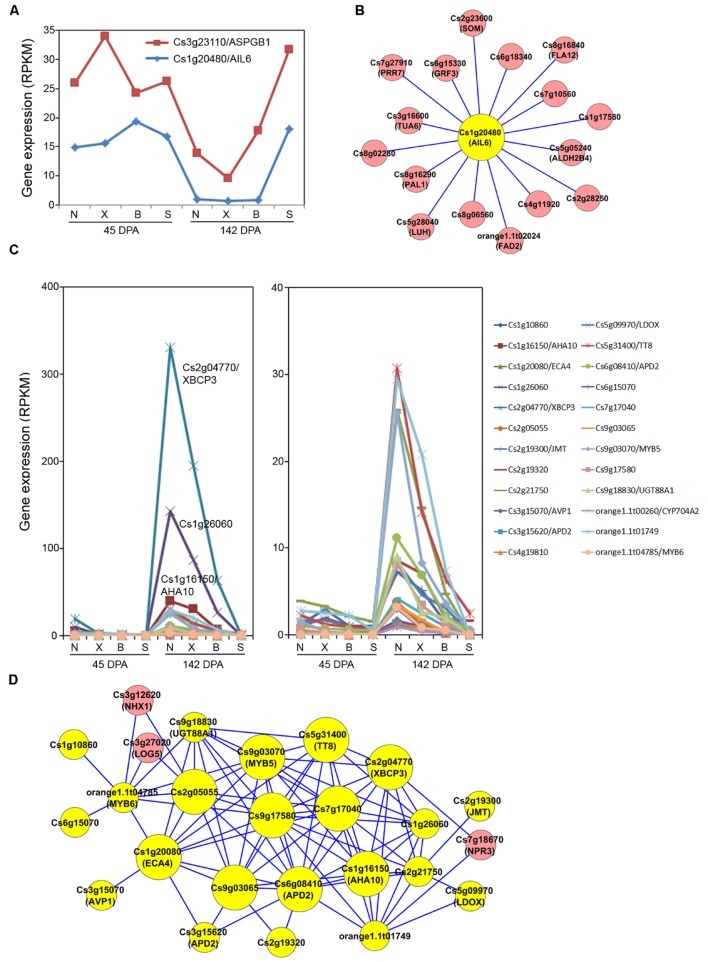
**Acid-related Magenta and Pink modules and subnetworks.** **(A)** Expression pattern for two of the acid-correlated genes from the Magenta module. N, X, B and S indicate Newhall, Xinhui, Bingtang and Succari, respectively. DPA, days post anthesis. **(B)** Subnetwork of the acid-correlated genes from the Magenta module. **(C)** Expression pattern for 24 of the acid-correlated genes from the Pink module. **(D)** Subnetwork of the acid-correlated genes extracted from the Pink module. The subnetworks are constructed by using the acid-correlated genes from the Magenta and the Pink modules, respectively, as seed nodes to extract the weighted gene coexpression network of all 7,430 differentially expressed genes. The resulting correlation subnetworks (edge weight larger than 0.2) are visualized by Cytoscape. The acid-correlated genes used as seed nodes are coded in yellow.

To provide a systems view of gene networks for the orange fruit acid control, networks from the three modules strongly correlated with acid levels, Magenta, Pink and Turquoise, were extracted. We first constructed the acid-correlated Magenta subnetwork using the two genes strongly associated with acid levels, Cs3g23110/ (*ASPGB1*) and Cs1g20480 (*AIL6*) as the seed nodes. The expression pattern for these two genes is shown in **Figure 5A**. While their expression in Newhall, Xinhui and Bingtang was down-regulated at the peak of acid accumulation (142 DPA) compared to 45 DPA, they maintained similarly high expression levels at 142 DPA in acidless Succari, showing a clear negative correlation with acid accumulation (**Table 2**). In this subnetwork, 16 genes had interactions with AIL6 (**Figure 5B**), although none of the genes showed strong interactions with ASPGB1. Among the AIL6-ineeracting genes, 15 have their closest homologs identified in *Arabidopsis* and nine of them are given specific gene names. For example, there are two genes likely involved in structural constituent (Cs8g16840/FLA12, a plasma membrane-associated FASCICLIN-like arabinogalactan-protein; and Cs3g1660/TUA6, an alpha tubulin), and four genes encode enzymes involved in various metabolic processes, including orange1.1t02024/FAD2 (fatty acid desaturase), Cs5g05240/ALDH2B4 (a mitochondria and chloroplast-localized NAD aldehyde dehydrogenase), and Cs8g1629/PAL1 (a Phe ammonia lyase). Most interestingly, five genes are likely involved in regulation of transcription, including two transcription factors (Cs2g23600/SOM and Cs1g17580) and three transcriptional regulators (Cs6g15330/GRF3, Cs7g27910/PRR7 and Cs5g28040/LUH). SOM (SOMNUS) is a CCCH-type zinc finger protein. PRR7 (PSEUDO-RESPONSE REGULATOR 7) is a two-component response regulator involved in light signaling and circadian rhythmic response (Kaczorowski and Quail, 2003). GRF3 (GROWTH-REGULATING FACTOR 3) is one of nine GRF-type transcription activators. LUH (Leuning-homolog) encodes a WD40 repeat and LUFS domain-containing protein which has a role in flower development (Sridhar et al., 2004). This subnetwork analysis indicates a possible transcriptional network involving the acid-correlated AIL6 transcription actor and other transcription factors or regulators.

We then analyzed the Pink module which contains the majority of the acid-correlated genes (**Figure 4B**). These 24 genes exhibit a pattern (**Figure 5C**) opposite to that in the Magenta module (**Figure 5A**), i.e., they showed up-regulation at 142 DPA compared to 45 DPA. These genes also show differential down-regulation between Bingtang and Succarri, and thus they are most strongly and positively correlated with acid accumulation in orange fruits. Among these genes, Cs2g04770/XBCP3, Cs1g26060, and Cs1g16150/AHA10 showed the largest changes in absolute expression levels in Bingtang and Succari. Gene coexpression network constructed by extracting these 24 genes from the Pink module revealed that most of these 24 genes are highly interconnected, with only two genes (Cs4g19810 and orange1.1t00260/CYP704A2) not present in this subnetwork and three genes which are not strongly correlated with acid (NHX1, LOG5 and NPR3; highlighted in pink) having interactions with the 22 acid-correlated genes (**Figure 5D**). In this subnetwork, 15 acid-correlated genes have 9–15 interactions and the remaining seven genes have only 1–3 interactions. The 10 genes having at least 10 interactions are labeled with larger node sizes in the subnetwork (**Figure 5D**). Among these, Cs6g08410/APD2, the most strongly acid-correlated gene has the largest number (15) of interactions. Cs3g15620, which also has a very strong correlation with acid level (**Table 2**) and is also most closely related to APD2, has only three interactions including the one with Cs6g08410/APD2. Most interestingly, Cs6g08410/APD2 gene has interactions with all the other nine hub genes with at least 10 interactions (**Figure 5D**). Two of them are genes encoding ATPases involved in proton (Cs1g16150/AHA10) or cation transport (Cs1g20080/ECA4). ECA4 is also connected to another proton transport protein Cs3g15070/AVP1. In addition, two transcription factor genes, Cs5g31400/TT8 and Cs9g03070/MYB5, are also connecting to Cs6g08410/APD2. These two transcription factor genes are connected to each other and each has interactions with other genes highly correlated with acid level. Moreover, MYB5 is also connected to orange1.1t04785/MYB6. The other functionally known hub gene, Cs2g04770/XBCP3, has interactions with APD2, TT8, MYB5, AHA10, ECA4, and others. *Arabidopsis* APD2 is a RING/U-box superfamily protein localized in intracellular membranes and recently shown to possess an E2-dependent E3 ligase activity in vitro (Luo et al., 2012). The cysteine peptidase XBCP3 in *Arabidopsis* is localized in vacuole and ER and predicted to be involved in proteolysis (Carter et al., 2004). Taken together, analysis of this Pink module-based acid-correlated subnetwork indicates that the three groups of genes involved in protein degradation, proton/calcium transport and transcription have a highly orchestrated transcriptional regulatory pattern.

**FIGURE 6:**
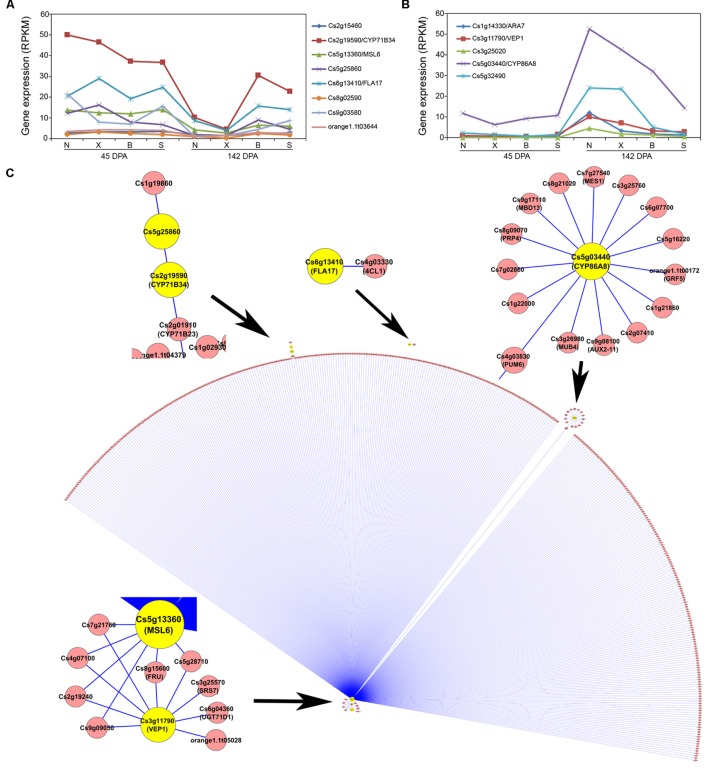
**Acid-correlated Turquoise module and subnetwork.** **(A)** Expression pattern for eight of the acid-related genes from the Turquoise module. N, X, B, and S indicate Newhall, Xinhui, Bingtang, and Succari, respectively. DPA, days post anthesis. **(B)** Expression pattern for five of the acid-related genes from the Turquoise module. **(C)** Subnetwork of the acid-correlated genes from the Turquoise module (edge weight larger than 0.2). Genes belonging to the Turquoise module are coded in yellow.

For the Turquoise module which contains 13 acid-correlated genes (**Figure 4B**), although the correlation between the Turquoise module eigengene expression and fruit acid levels is negative (–0.59; **Figure 4B**), these 13 genes actually exhibit two contrasting patterns (**Figures 6A,B**). One is represented by eight genes which exhibit strong down-regulation in Newhall and Xinhui but weak in Bingtang and Succari (**Figure 6A**), and the other represented by five genes which show strong up-regulation in Newhall and Xinhui but weak in Bingtang and Succari (**Figure 6B**). For the first pattern (**Figure 6A**), which has negative correlations with acid level (**Table 2**), Cs2g19590/CYP71B34 and Cs6g13410/FLA17 show the most dramatic expression differences. For the second pattern (**Figure 6B**), which has positive correlations with acid level (**Table 2**), Cs5g03440/CYP86A8 and Cs5g32490 display the largest difference in expression levels. Using these 13 acid-related genes as the seed nodes to extract the Turquoise module, a huge subnetwork was derived (**Figure 6C**). This subnetwork contains six of the acid-correlated genes (highlighted in yellow) plus 412 other genes, with a total of 421 interactions (**Supplementary Figure S3** and **Table S3**). However, the majority (393) of these genes have the interactions with Cs5g13360/MSL6, the largest hub in the subnetwork (**Figure 6C**). Among those 393 genes, six of them connect MSL6 to another acid-correlated gene, Cs3g11790/VEP1. In addition, Cs4g03830 (most closely related to *Arabidopsis* PUMILO 6 or PUM6) connects MSL6 to a small hub, Cs5g03440/CYP86A8, another acid-correlated gene (**Table 2**). Indeed, another cytochrome P450-type gene, Cs2g19590/CYP71B34, is also present in this subnetwork. MSL6 has a relatively weak, negative correlation (Pcc = –0.80; **Table 2**) with acid level and down-regulation at 142 DPA is weaker in Bingtang and Succari than that in Newhall and Xinhui (**Figure 6A**). *Arabidopsis* MSL6 is a member of mechanosensitive ion channel proteins likely involved in sensing and responding to mechanical stimuli such as touch, osmotic pressure (Haswell et al., 2008).

### SVA of Candidate Genes Related to Fruit Acidity

After controlling the effects of genotypes, developmental stage and unmodeled latent factors, we identify a total of 104 genes that are significantly associated with the acid level, using an FDR cutoff at 0.0005 (**Supplementary Table S4**). Venn diagram analysis shows that 15 of those genes (**Table 3**) overlap with the list of 39 genes (**Table 2**) identified using Pearson correlation analysis. Among these 15 genes, 11 belong to the Pink module, one the Magenta and three the Turquoise modules (**Table 3**). Interestingly, the majority of these genes are the hub genes in the acid subnetworks (**Figures 5** and **6**), and all three genes involved in protein degradation (Cs6g08410/APD2, Cs3g15620/APD2, and XBCP3) and three of five in transport process (AHA10, ECA1 and Ara7) are revealed by all three distinct algorithms (Pearson, WGCNA, and SVA).

**Table 3:** A list of 15 acid-related candidate genes identified by three algorithms.

CsGID	Gene Name	SVA (FDR)	Pearson correlation (Pcc)	WGCNA modules
Cs6g08410	APD2	1.6E-04	0.96	Pink
Cs3g15620	APD2	3.7E-04	0.93	Pink
Cs2g04770	XBCP3	3.8E-04	0.85	Pink
Cs1g16150	AHA10	3.1E-05	0.89	Pink
Cs1g20080	ECA4	3.6E-06	0.93	Pink
Cs9g18830	UGT88A1	3.2E-04	0.89	Pink
Cs2g19300	JMT	4.5E-04	0.87	Pink
orange1.1t04785	MYB6	3.2E-04	0.85	Pink
Cs9g17580		4.4E-04	0.93	Pink
orange1.1t01749		1.8E-04	0.90	Pink
Cs1g26060		4.7E-04	0.87	Pink
Cs1g20480	AIL6	7.3E-06	-0.80	Magenta
Cs1g14330	ARA7	3.7E-04	0.84	Turquoise
Cs2g19590	CYP71B34	1.3E-05	-0.88	Turquoise
Cs6g13410	FLA7	2.8E-04	-0.81	Turquoise


*A total of 15 genes are present in both the list of 39 acid-correlated genes (**Table 2**) identified using Pearson correlation analysis and the list of 104 genes (**Supplementary Table S5**) identified by Surrogate Variable Analysis (SVA). The majority of these genes are in the Pink module revealed by WGCNA (weighted gene coexpression network analysis). CsGID, Cs gene ID; FDR, false discovery rate; Pcc (Pearson correlation coefficient).*

To further validate the result, we used qRT-PCR to analyze the expression of Cs1g16150/AHA10 and Cs6g08410/APD2, two genes from the list (**Table 3**), in fruits of 142 DPA collected from a different year than the year for RNA Seq analysis. Our results showed that expression of these two key hub genes in the Pink module (**Figure 5D**) was significantly correlated with the RNA Seq data (**Supplementary Table S5**). Taken together, this subset of 15 genes revealed through our integrated systems analysis may represent the candidate genes most likely associated with fruit acidity in developing orange fruits.

## Discussion

Because of the paramount importance of manipulating fruit acidity in improving fruit quality, many genetic and transcriptomic studies have been performed with a goal of unraveling genetic mechanisms of citrate and malate accumulation in fruits. Except one melon study (Cohen et al., 2014), most of forward genetic studies have not revealed the genes that show causative effects in fruit acidity control. Forward genetic study in citrus is severely hindered because of its extremely complex genetic background and long juvenile phase. We therefore addressed this key question by using an integrated systems biology approach.

### Our Unique Transcriptome and Systems Biology Study Has Led to the Identification of a List of Candidate Genes Likely Associated with Fruit Acidity Control

Although a number of transcriptomic studies in tomato, orange and other fruits have been reported, our transcriptome profiling and network analysis differ from those prior studies in several ways. First, several of the prior transcriptomic studies used the RNA samples from fruits of normal acidity or the ripening mutants showing pleiotropic phenotypes (Alba et al., 2005; Cercos et al., 2006; Janssen et al., 2008; Mounet et al., 2009; Fasoli et al., 2012; Osorio et al., 2012; Kang et al., 2013). Second, in case that acidless varieties were included, these studies did not analyze the samples at Stage I or early stage II which had similar acid levels for both acidic and acidless varieties and thus it remains a challenge to have a sound comparison of gene expression (Aprile et al., 2011; Yu et al., 2012; Wu J. et al., 2014). In our study, three types of sweet orange fruits have been used for comparison, acidless variety (Succari), low acid variety (Bingtang), and two varieties with normal acid levels (Newhall and Xinhui). Furthermore, we have compared the fruit samples at late Stage II (where citrate levels exhibit a large difference between the varieties) with those close to the end of Stage I (where citrate levels do not differentiate between the varieties). Consequently, we are able to identify a list of 39 genes strongly associated with acid levels, among a large number (7,430) of genes which exhibited differential regulation from Stage I to Stage II in any of the four sweet orange varieties. Third, we have integrating the results using three different algorithms (Pearson, WGCNA and SVA) to identify a total of 15 genes (**Table 3**) which are found to be most likely associated with fruit acidity. In addition, the majority of these 15 genes are hub genes in the acid accumulation gene networks. Clearly, use of these four sweet orange genotypes with varying fruit acidity and through integrated systems analysis have led to the identification of candidate genes that are small in number but likely more specifically associated with the control of organic acids in developing fruits.

### Three Distinct Regulatory Modules Provide Novel Insights into the Orange Fruit Acidity Control

To provide clues to the possible involvement of citrate synthesis and GABA shunt in the fruit acidity control during early stages of sweet orange development, we examined the expression of genes predicted to function in citrate synthesis or GABA shunt. We did observe that some of *Aco1/2/3* and *GAD1/2* genes encoding enzymes critical for citrate utilization and GABA shunt (in particular for *Aco3* and *GAD2*) were up-regulated from 45 to 142 DPA (**Supplementary Table S6**). However, the expression difference at 142 DPA between four varieties with varying acidity was either lacking or at most marginal (less than twofold), in sharp contrast to prior studies (Terol et al., 2010; Aprile et al., 2011; Liu et al., 2014). In addition, the genes encoding citrate synthase (orange1.1t01588 and Cs7g01170) did not exhibit any expression difference between 45 and 142 DPA and among four varieties, and the genes predicted to function as putative citrate synthase (such as Cs7g08950 and Cs9g02230; **Supplementary Table S1**) decreased expression slightly from 45 to 142 DPA but similarly in all four varieties. On the other hand, none of the 39 acid-correlated genes identified by Pearson correlation analysis encodes enzymes directly involved in citrate synthesis and the proposed citrate utilization model including the GABA shunt. Although we cannot exclude the possibility that those genes involved in citrate synthesis or GABA-shunt can still be regulated at the translational or post-translational level, our result raises a possibility for a citrate synthesis and GABA shunt-independent mechanism at the transcriptional level in maintaining low accumulation of citrate at Stages I and II at least in the two sweet orange varieties analyzed here, Bingtang and Sucarri.

Interestingly, our systems analysis has revealed the existence of three distinct regulatory modules, Magenta, Pink and Turquoise. While the Magenta module of the network contains genes that show negative correction, the major module Pink (with 24 of the acid-correlated genes) is positively correlated with acid levels, and the Turquoise module exhibits both positive and negative correlations. Among these three modules, the Pink might play a predominant role in the control of fruit acidity. This is because more than 60% of the acid-related genes belong to this module, and this gene number represents 47% of all the Pink module genes in the fruit development network. In addition, gene coexpression network analysis suggests that transcription factors may act as major hubs in the acid control subnetworks, such as AIL6, TT8, and MYB5. The other type of acid-correlated genes can be grouped into the GO categories of transport and protein degradation. The transport category includes AHA10, ECA4, AVP1, MSL6, and ARA7. The first three belong to the Pink module and they connect with each other and with other proteins in the subnetwork, while the latter two genes belong to the Turquoise module. MSL6 turns out to be a super hub, interacting with almost 400 genes. AHA10 and AVP1 are shown to act as proton pumps in *Arabidopsis*, consistent with a biochemical study where two types of H^+^-ATPases activities are found in vacuoles of lemon fruits (Muller et al., 1996). The most interesting one is AHA10. Correlation of *AHA10* expression with fruit acidity has been reported by an earlier study which compared acidic and acidless lemon fruits (Aprile et al., 2011) and was recently confirmed in orange and pummelo varieties with differential acid levels (Shi et al., 2015). Together with our finding, we hypothesize that AHA10 might represent a conserved regulatory molecule involved in acid accumulation in the vacuole of sweet orange and lemon fruits. Indeed, convincing genetic evidence has demonstrated that *Arabidopsis* AHA10 is involved in vacuolar biogenesis and pH regulation (Baxter et al., 2005). The study of vacuolar acidification in *Arabidopsis* also led to the finding that anthocyanin accumulation is subjected to the regulation by AHA10. Consistent with this finding, our study has also revealed that one of the positively acid-correlated genes, Cs5g09970, is most similar to *Arabidopsis* LDOX involved in proanthocyanin biosynthesis and vacuole formation (Abrahams et al., 2003). Therefore, our work raises an intriguing possibility that AHA10-type ATPase has a conserved function in controlling pH or acidity in fruits or other organs in many types of plants. It will be interesting to test this hypothesis in fruit crops such as orange and tomato.

Another potentially interesting result from our work is the identification of regulatory proteins involved in protein degradation. This includes one XBCP3-like cysteine peptidase and two genes most closely related to *Arabidopsis* APD2, a RING/U-box superfamily protein involved in ubiquitin-mediated protein degradation (Luo et al., 2012). They all belong to the Pink module and are identified by the other two algorithms, Pearson and SVA. These two APD2-like genes exhibit very strong correlations with acid levels, with the most strongly correlated one (Cs6g08410) acting as a major hub in the subnetwork by interacting with transcription factors (TT8 and MYB5) and ion pumps AHA10 and ECA4. Mutants of *APD2* and three other closely related *APD* genes have been reported to alter pollen development due to a defect in cell division (Luo et al., 2012), and thus it remains unclear whether these genes are also involved in fruit development and/or acid control in orange. Of particular note, a preliminary yeast two-hybrid study using *Arabidopsis* APD2 as a bait resulted in the identification of two proteins, AT1G75630 and AT2G25610, which are parts of subunit C of the V-type ATPase that catalyzes ATP hydrolysis to transport protons (Luo et al., 2012). Taken together, the results from our integrated systems analysis together with insights from studies in the *Arabidopsis* model system provide a testable intriguing hypothesis that the ubiquitin-mediated protein degradation pathway might link ion transport and transcription factors in the control of citrate accumulation in orange fruits.

In summary, our integrated systems analysis of the acid-related genes has led us to propose interesting hypotheses involving ion transport, protein degradation and transcription for the genetic control of citrate accumulation in orange fruits. As in almost all of the transcripomic studies, it remains a daunting task to determine which of those candidate genes actually have regulatory roles in the fruit acidity control. A recent study in *Arabidopsis* seed germination reported that 22–50% of the hub genes in the seed network turned out to have physiological functions in seed germination (Bassel et al., 2011). Therefore, our integrated systems biology analysis provides an important basis for future study of those hub genes identified here to test whether they play important roles specifically in citrate accumulation or broadly in fruit development in citrus which has a potential to be used as a model for non-climacteric fruits.

## Author Contributions

Z-LZ conceived of the project, designed the experiments, performed bioinformatic analysis, analyzed the data, and wrote the manuscript; DH performed the RNA extraction and qPCR validation experiments, and assisted in writing; YH performed bioinformatic and statistical analyses, and wrote the manuscript; and MC and LQ performed the qPCR validation and repeated the experiment, and assisted in writing.

## Conflict of Interest Statement

The authors declare that the research was conducted in the absence of any commercial or financial relationships that could be construed as a potential conflict of interest.

## Footnotes

1 http://citrus.hzau.edu.cn/orange/

## Supplementary Material

**FIGURE S1 | Fruit growth of four sweet orange varieties.** The three stages of fruit development were separated by dotted lines, and the two time points were shown for which fruit sugar and acidity were measured and transcriptomes analyzed.

**FIGURE S2 | Cluster analysis of 7,430 genes.**

**FIGURE S3 | A fully expanded view of the Turquoise acid subnetwork shown in Figure 6C.** The PDF file can be enlarged to view the details of gene IDs in the network.

**TABLE S1 | A list of 7,430 differentially expressed genes from 45 to 142 DPA and 20 clusters represented by these genes.**

**TABLE S2 | A list of primers used for qRT-PCR analysis of seven selected genes.**

**TABLE S3 | A list of acid-correlated genes in the Turquoise module used in the acid subnetwork construction.**

**TABLE S4 | A list of 104 acid-related genes identified using Surrogate Variable Analysis (SVA) approach.**

**TABLE S5 | Pearson correlation analysis of AHA10 and APD2 expression levels detected by RNA sequencing and qRT-PCR analysis of an independent repeat.**

**TABLE S6 | Expression levels of Aco and GAD genes in fruits collected from another year.**
