# Supplementary figures and images for: Integrated Systems Biology Analysis of Transcriptomes Reveals Candidate Genes for Acidity Control in Developing Fruits of Sweet Orange (Citrus sinensis L. Osbeck)
Source: Front Plant Sci. 2016 Apr 8;7:486. doi: 10.3389/fpls.2016.00486 (PMC4824782; doi:10.3389/fpls.2016.00486)

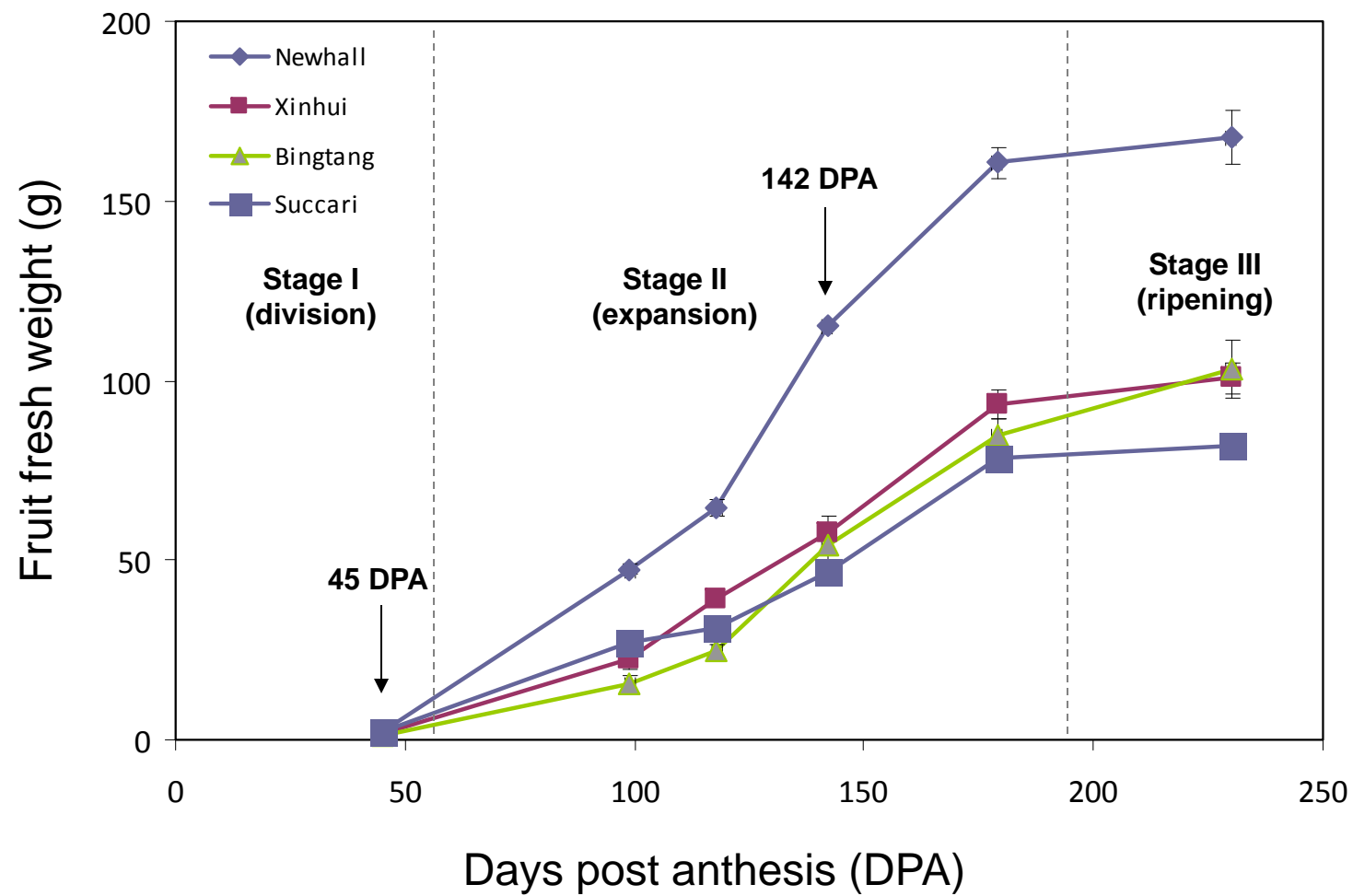

Supplement: Supplementary file 1 [file Image_1.PDF]

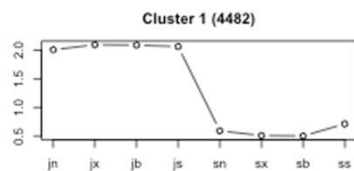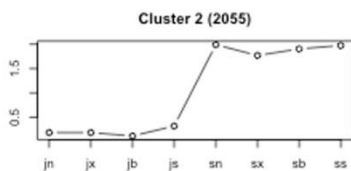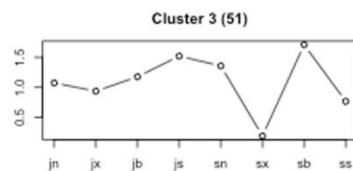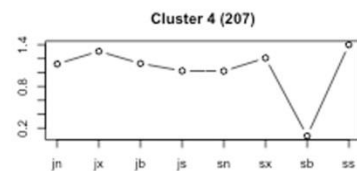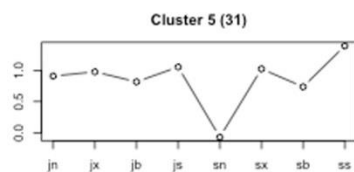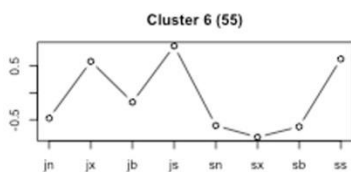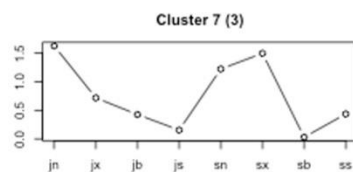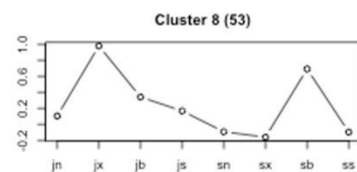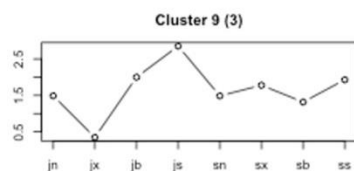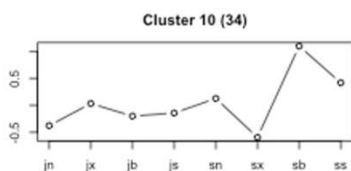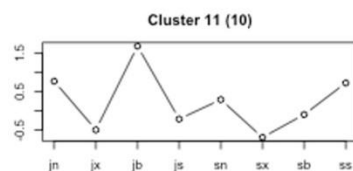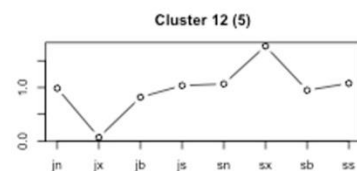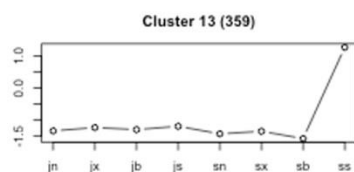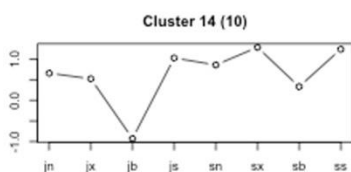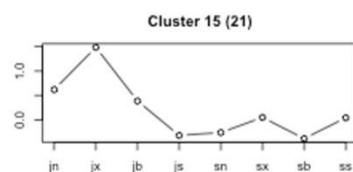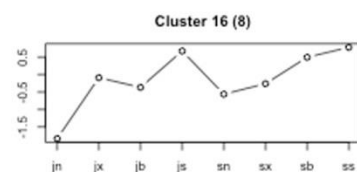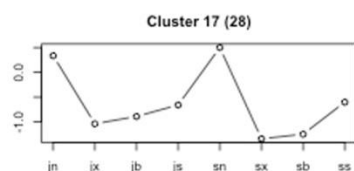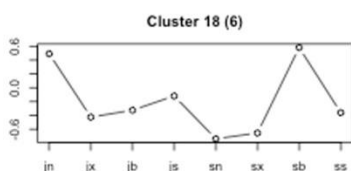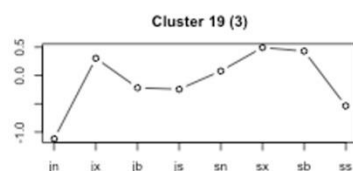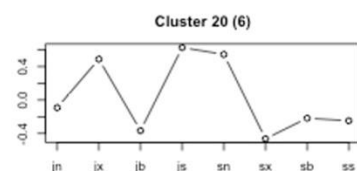

Supplement: Supplementary file 2 [file Image_2.PDF]

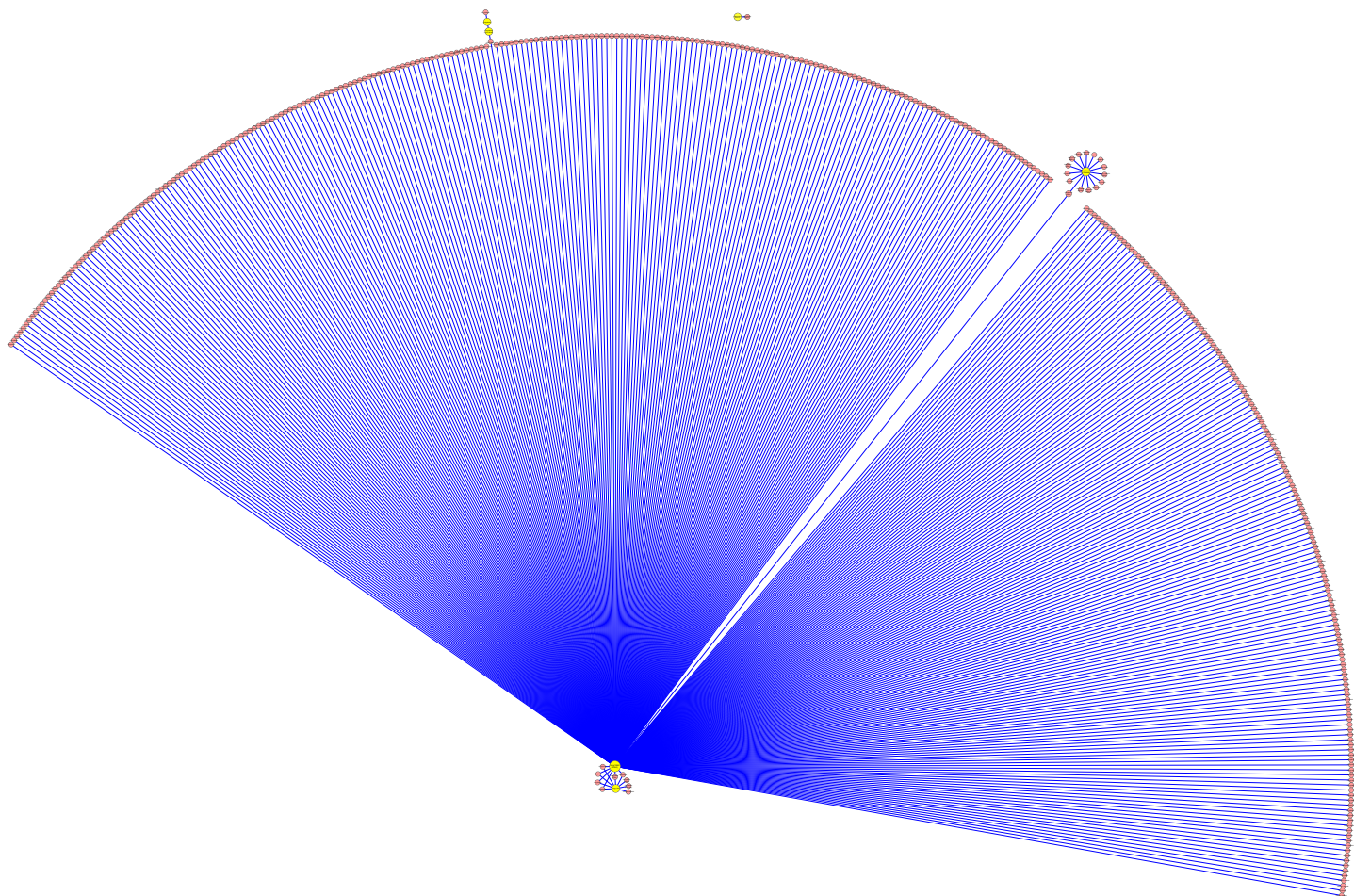

Supplement: Supplementary file 3 [file Image_3.PDF]
